# Motivation for the Family Visit and On-the-Spot Activities Shape Children’s Learning Experience in a Science Center

**DOI:** 10.3389/fpsyg.2021.629657

**Published:** 2021-04-07

**Authors:** Pirko Tõugu

**Affiliations:** Institute of Psychology, University of Tartu, Tartu, Estonia

**Keywords:** informal learning, parent-child conversation, science centers, encouraging experimentation, motivation

## Abstract

Children’s learning often happens in the interactions with more knowledgeable members of the society, frequently parents, as stated by the sociocultural theory. Parent-child conversations provide children with a new understanding and foster knowledge development, especially in informal learning contexts. However, the family conversations in museums and science centers can be contingent on the motivation for the family visit or the activities organized on the spot. In order to establish how family motivation and on-the-spot activities influence children’s informal learning experience, the present study was carried out in a family science center. The study focused on children’s learning experience in a hands-on exhibit featuring objects that allow for the exploration of the concepts of sound waves and light. Thirty-nine 7–10-year-old children (21 boys and 18 girls) and their families participated in the study. Twenty families received a worksheet to prompt an experimentation activity with one of the light exhibits. Motivation for the family visit was probed at the end of the visit. The target children of the families wore a GoPro HERO 5 camera attached to a chest harness throughout their visit. The video was coded for family interaction and experimentation with the light exhibit. Family conversations were coded for open-ended questions, responses to open-ended questions, explanations, associations, attention directing, and reading signage aloud. Family motivation for the visit was related to the quality of family conversation during the visit. The experimentation activity prompt did not affect the likelihood of noticing and engaging with the particular exhibit. At the same time, it did affect the quality of engagement: children who received the experimentation activity prompt were more likely to explore the effects the exhibit provided and experiment rather than play with the exhibit. Family motivation and on-the-spot activities are discussed as two possible factors to influence children’s learning experience in science centers.

## Introduction

Museums and other informal learning institutions provide a unique engaging learning space for children and families. Museums nowadays embrace their role as educational agents for children and often keep children and families in mind when designing exhibits and providing interactive and hands-on learning opportunities (Allen, 2004; Heath et al., 2005; Müller, 2013). When families visit informal education institutions with children, learning most often takes place when parents scaffold the experience providing structure and helping children to make sense of the learning opportunities (Haden, 2010; Andre et al., 2017). Parents’ motivation to do so, on the one hand, and the structure provided by the museum (i.e., exhibit design, on-the-spot activities, additional material and signage, etc.), on the other, could influence the resulting experience of children. The present study focuses on family visits to a science center to establish how parent’s motivation for the visit and the on-the-spot activity relate to family interaction and exploration that shape children’s learning experience.

Prior research has identified parent-child interaction in informal settings as the mechanism for learning (Crowley et al., 2001a; Fender and Crowley, 2007; Benjamin et al., 2010; Rigney and Callanan, 2011; Andre et al., 2017). Such research is rooted in the sociocultural theory stating that learning mostly happens in activities, where children engage and interact with more knowledgeable members of the society (Vygotsky, 1978). In parent-child interaction, several structural elements have been identified as cognitively demanding science talk that improves children’s understanding of the world and helps them to construct knowledge. These structural elements include questions, explanations, use of analogies and associations, and suggestions to test hypothesis (Crowley et al., 2001b; Tenenbaum and Leaper, 2003; Tenenbaum et al., 2005; Valle and Callanan, 2006; Tare et al., 2011). Parents’ questions and associations to prior knowledge have also been linked to children’s improved learning in the museum and memory for the experience (Benjamin et al., 2010).

In informal learning institutions, the family conversations, in particular the explanations, associations, and open-ended questions, often go hand in hand with the exploration of the exhibit, both supporting learning and meaning making (Gutwill and Allen, 2010; Callanan et al., 2020). Different exhibits have different affordances for exploration. Studies seem to suggest that adding an opportunity to manipulate objects could be engaging and even beneficial for the children of the visiting family. For example, Jant et al. (2014) have shown that an opportunity to manipulate objects accompanied by prompts to parents to ask wh-questions leads to richer parent-child interaction and improved learning and memory for the experience. At the same time, the exploration of hands-on displays could give rise to more learning opportunities when children receive some scaffolding of experience. Crowley et al. (2001a) showed that children who studied a hands-on exhibit with a parent had a broader and more focused exploration as parents explicitly made connections and provided explanations to help children understand the phenomena. This seems to suggest that providing some structure for children’s free exploration of exhibits could be advantageous for their learning experience.

Different on-the-spot activities in the museum are often successful in creating enhanced learning opportunities. The activities to support such learning can be very different and the examples are manifold. For example, engaging families in play with numerical prompts leads to more spontaneous focus on numbers afterward (Braham et al., 2018). Inquiry games, especially the ones that involve the whole family, have been shown to deepen the conversations and learning at a science museum (Gutwill and Allen, 2010). Instructing parents to either encourage exploration or explanation with their children results in longer explorations or more discussion, respectively (Willard et al., 2019). Providing families with topical activities increases talk on the topic in the exhibit (Tenenbaum et al., 2010). Providing exhibit relevant information to parents and inviting them to use open-ended questions brings about more science, technology, engineering, and math (STEM) talk focused conversations during the museum activities and more STEM talk in recollections of the experience (Benjamin et al., 2010; Haden et al., 2014; Marcus et al., 2017). Adding questions to signage boosts metacognition (Gutwill and Dancstep, 2017) and family conversations (Land-Zandstra et al., 2020). All in all these intervention studies seem to indicate that providing some structural support or guidance to otherwise open-ended museum visits could magnify the learning opportunity for the children as it brings about deeper and more focused conversations.

Motivation is closely related to learning in general, and learning in informal settings is no exception. Visitors may come to the museum for a large variety of reasons, e.g., social reasons, interest in the subject, entertainment etc. (Falk et al., 1998; Rowe and Nickels, 2011; Ji et al., 2014). Motivation for the museum visit has been shown to shape the informal learning experience for adults and to affect their learning outcome (Falk et al., 1998; Moussouri and Roussos, 2013). Adults with a learning motivation remember more concepts at the end of the visit as compared to adults without such motivation (Falk et al., 1998) and spend their time mostly visiting exhibits rather than socializing (Moussouri and Roussos, 2013). There is not any information available about how parents’ motivation for the museum visit relates to children’s experience and learning.

### The Present Study

The present study was carried out in The Energy Discovery Center in Tallinn, Estonia. It is a family science center, where one can discover, play, and learn (www.energiakeskus.ee). The center is popular with families and engaging for children with their hands-on exhibits to discover the principles of electricity, light, sound, and other physics phenomena. The present study focused on children’s learning experience in a hands-on exhibit featuring objects that allow for the exploration of the concept of sound waves and light.

The aim of the study was to establish how parental motivation for the visit and the on-the-spot experimentation activity prompt relate to the behavior and conversations of the family at the exhibit. Prior research has consistently shown that on-the-spot interventions and activities successfully shape the learning experience of the family and children. Therefore, it was expected that providing a specific experimentation activity prompt would elicit more engagement and experimentation with the exhibit. In addition, family motivation for the science center visit could affect their behavior in the center. In particular, families with a learning motivation could engage in different learning and teaching behaviors more often than families with a different focus.

## Materials and Methods

### Participants

Forty children (22 boys and 18 girls) with their families participated in the study. The average age of the participating children was 8 years (range 7–10). In 19 cases, the child visited the science center with one parent, and in other cases, there were two or more adults with the family. Eight children were only children in the family group; the rest of the families had siblings along for the visit. For 31 families, this was the very first visit to The Energy Discovery Centre; nine families had visited the center before. Additional demographic data (e.g., socioeconomic status) about the families was not collected. Due to a technical mishap, video data of one participant are missing, resulting in full data about the visit of 39 families. In the analyses, the data of the family are deleted listwise.

### Procedure

Participants were recruited as they entered the exhibit: families with children in the age range of 7–10 years were approached and invited to participate in the study. Consent to participate in research was obtained from adults and children. Once children agreed, a GoPro HERO 5 camera was attached to them using the Junior Chesty chest mount. All the families were told to take their time and explore the exhibit as they normally would. By random assignment, half of the participants (*n* = 20) also received a worksheet with an experimentation activity prompt. They were told to see if they can find answers to the prompts on the worksheet during their visit and asked to fill it during their exploration. Other than that, their instruction was identical to the families not receiving the experimentation activity prompt. After the visit, as the family was ready to leave, the parent filled in a short questionnaire providing background information, including the motivation (recreation, fun, to learn something new etc.), for their visit.

### Measures

#### Motivation

Motivation for the family visit was probed at the end of the visit with a questionnaire. Five possible motivations (e.g., to have fun, to learn something new, etc.) and an option to fill in their motivation for the visit was listed, and the parent was instructed to indicate up to three.

#### Experimentation Activity Prompt

The experimentation activity prompt focused on the concept of shadow that was featured in one of the exhibits in the center (Shadow theater). The prompt contained a playful task to indicate what is necessary for the shadow to appear (children had to circle the necessary objects), and two multiple choice questions that could sprout experimentation: what happens to a shadow when (a) the object is moved closer to the light and (b) when the object is turned?

### Coding

#### Conversations

The videos captured with GoPro HERO 5 were coded using the Noldus Observer XT program. The coding scheme was the same for adults and children. In order to reveal the learning conversations of the families, the following types of instances were coded as they occurred:

- Directing attention: utterances that directed the other person’s attention to a particular exhibit or an aspect of the exhibit, e.g., “*Look at this!*”; “*Hear this!*”; “*You should look at this here!*”; and “*The light goes here, see!*”
- Open-ended questions: wh-questions asking about a particular exhibit or an aspect of the exhibit, e.g., “*What does this do?*”; “*What do you see?*”; and “*What happens when you do that?*”
- Responses to open-ended questions: verbal responses to the wh-questions about particular exhibits or aspects of the exhibit.
- Explanations: utterances that went beyond describing the objects and focused on why a particular phenomenon occurred or how an exhibit displayed the particular phenomenon, e.g., “*The light deceives you, see, these edges make it look like it is much deeper than it actually is*” and “*See, these are solar panels on the wings -- light turns into energy there and makes the propeller move.*”
- Reading aloud: instances of reading the signage at the exhibits aloud either to themselves (children) or to the children (adults).
- Associations: e.g., “*This is like a small earthquake!*” (about thunder soundwaves) and “*This is like the solar panels our house has.*”

The conversational codes were assigned to the children and adults of the family. All conversations that included the target child were coded including conversations with other family members and siblings. Two people coded 15% of the videos; the interrater agreement Kappa was 0.78 on the average for parents’ conversational codes and 0.76 for children’s conversational codes with no single value below 0.7. Disagreements were resolved in discussion and the author proceeded to code the rest of the videos.

#### Child Engagement With the Experimentation Activity Exhibit

**Figure 1:**
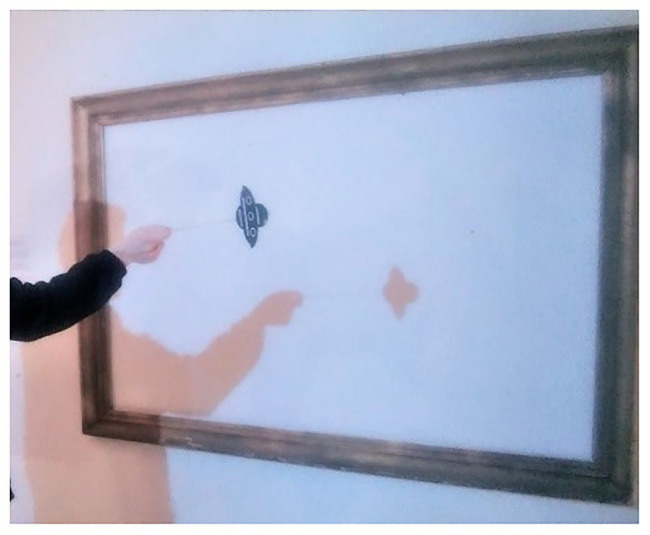
The Shadow theater.

The experimentation activity prompt included open-ended questions that could be explored at the shadow theater exhibit (Figure 1). In order to investigate children’s engagement with the particular exhibit, two aspects were coded. First, it was coded whether the children noticed the shadow theater or not. Noticing the exhibit was coded when either (a) the child or parent picked up the trafarets and placed them against the screen or (b) tried to make shadow animals on the screen (as also shown on a signage at the exhibit) or (c) verbally commented on the shadow theater. Secondly, it was coded whether the children or parents experimented and explained the qualities of the shadow, moving the trafarets closer and further or turning them sidewise to show how the shadow changed. Two people coded the engagement with the experimentation activity exhibit from the video for 15% of the videos. There were no disagreements and the author proceeded to code the rest of the videos.

## Results

Families spent 38 min (*SD* = 12, range 18–69 min) on average in the hands-on exhibit at the science center. The length of the visit did not differ for boys and girls or families with and without the experimentation activity prompt or for the families who reported the learning motivation as compared to those who did not. Correlation analysis revealed that family size was not related to the length of the visit, but it was negatively associated with some of the parental conversational variables, namely open-ended questions, associations, attention directing, and reading signage aloud [*r*s = −0.32 – (−0.38), *p*s = 0.02–0.04].

### Motivation

Families were instructed to indicate three main motivations for the visit from a list of possible reasons. Families selected 1–4 motives (*M* = 2.68, *SD* = 1.05). Table 1 displays the reasons for the visit and the number of families that selected the reason. Chi-square analyses were run for the different types of motivation separately to see if they were related to the gender of the child or the fact that the family received the experimentation activity prompt. Different motivations reported by the adult were not related to child gender [*χ*^2^-s (1, *N* = 39) = 0.04–1.36, *p*s = 0.24–0.84]. Neither did the reported motivations differ for families who received the prompt and for those who did not [*χ*^2^-s (1, *N* = 39) = 0.44–1.91, *p*s = 0.17–0.51]. Motivation to learn something new was indicated by 16 families (40%), and this was used as a grouping variable for the analyses of the conversational codes.

**Table 1:** Family motivation for the visit.

Motivation	Number of families indicating the motivation	Percentage of families indicating the motivation
They have been here before and enjoy the particular science center	6	15%
They wanted to do something together	30	75%
They wanted to learn something new	16	40%
They wanted to have fun	26	65%
It seemed like an interesting place to visit	28	70%

### Conversations

Conversational codes were collapsed for children of the family and adults of the family. Many of the conversational codes were infrequent (see Table 2) and the distribution of all the conversational variables did not adhere to normal distribution. Therefore, median split was used to create categorical variables for all the conversational codes and define groups of children and adults who used the variable often as compared to those who rarely used the variable. For the variables with a median of 0 (see Table 2), groups were defined as children/adults who did not use the particular conversational variable vs. children/adults who used the conversational variable at least once. First, possible gender differences in the use of conversational variables were studied. Chi-square analysis showed a significant association between child gender and adult explanations (*χ*^2^(1) = 4.31, *p* < 0.05) and child gender and child attention directing (*χ*^2^(1) = 4.31, *p* < 0.05). Sixty-one percent of girls (11/18), whereas only 38% of boys (8/21), received at least one explanation from their parents. Girls were more likely to use attention directing more often: 67% of girls (12/18) and only 33% of boys (7/21) belonged to the group that used attention directing above the median. The other conversational variables were not associated with child gender [*χ*^2^-s (1, *N* = 39) = 0.08–2.92, *p*s = 0.09–0.81].

**Table 2:** Median and range for the total number of conversational codes for children and parents.

Conversational variables	Parents *Median (Min-Max)*	Children *Median (Min-Max)*
Responses to questions	2 (0–13)	1 (0–6)
Open-ended questions	2 (0–15)	7 (1–37)
Attention directing	9 (0–35)	10 (1–24)
Explanations	0 (0–15)	0 (0–2)
Associations	0 (0–8)	0 (0–2)
Reading aloud	1 (0–28)	0 (0–15)

For testing the hypothesis, three-way log-linear models including experimentation activity prompt (Yes/No) and learning motivation as reported by parent (Yes/No) were built for each of the conversational variable for parents and children. For the analysis of parent’s Open-Ended questions, the three-way log-linear model analysis produced a final model that retained the Motivation x Open-Ended Questions interaction. The likelihood ratio of this model was *χ*^2^(4) = 4.05, *p* = 0.40, and the Motivation x Open-Ended Questions interaction was significant, *χ*^2^(1) = 5.23, *p* < 0.05. The odds ratio indicated that the odds of parents asking more than two open-ended questions were 4.8 times higher when they also reported a learning motivation for the visit. The analysis of parent’s Responses to Open-Ended Questions produced a final model that retained the Motivation x Responses to open-Ended Questions interaction. The likelihood ratio of this model was *χ*^2^(4) = 4.05, *p* = 0.40, and the Motivation x Open-Ended Questions interaction was significant, *χ*^2^(1) = 5.23, *p* < 0.05. The odds ratio indicated that the odds of parents responding to more than two open-ended questions were 4.8 times higher when they reported a learning motivation for the visit. The count of parents with and without learning motivation using these conversational variables is provided in Table 3. The models for parent’s use of Directing Attention, Explanations, Associations, and Reading Aloud did not provide statistically significant results.

**Table 3:** Crosstabulation of adults who asked and answered two or less or more than two open-ended questions by their learning motivation.

Conversational variable	Groups	Number and % of adults reporting a learning motivation	Number and % of adults not reporting a learning motivation
Open-ended questions	Number of adults who asked more than 2	10 (62.5%)	6 (27.5%)
	Number of adults who asked 2 or less	6 (26%)	17 (74%)
Responses to open-ended questions	Number of adults who responded to more than 2	10 (62.5%)	6 (27.5%)
	Number of adults who responded to 2 or less	6 (26%)	17 (74%)

For the analyses of children’s Associations, the three-way log-linear model analysis produced a final model that retained the Experimentation Activity Prompt x Association interaction. The likelihood ratio of this model was *χ*^2^(4) = 4.72, *p* = 0.32, and the Experimentation Activity Prompt x Association interaction was significant, *χ*^2^(1) = 6.63, *p* < 0.05. The odds ratio indicated that the odds of children making an association were 11.6 times higher when they had not received the experimentation activity prompt. The analysis of children’s Explanations produced a final model that retained the Experimentation Activity Prompt x Explanations interaction. The likelihood ratio of this model was *χ*^2^(4) = 5.68, *p* = 0.22, and the Experimentation Activity Prompt x Explanations interaction was significant, *χ*^2^(1) = 4.12, *p* < 0.05. The odds ratio indicated that the odds of children providing explanations were 5.3 times higher when they had not received the experimentation activity prompt. The count of children with or without experimentation activity prompt using these conversational variables is provided in Table 4. Models for Directing Attention, Open-Ended Questions, Responses to Open-Ended Questions, Explanations, and Reading Aloud did not provide statistically significant results.

**Table 4:** Crosstabulation of children using explanations or not and receiving experimentation activity prompt or not.

Conversational variable	Groups	With prompt (*n*, %)	No prompt (*n*, %)
Associations	Number of children who made at least one	1 (12.5%)	7 (87.5%)
	Number of children who did not make any	19 (61%)	12 (39%)
Explanations	Number of children who used at least one	2 (22%)	7 (78%)
	Number of children who did not use any	18 (60%)	12 (40%)

### Child Engagement With the Experimentation Activity Exhibit

Most of the children and families (*n* = 32, 80% of all the families) noticed the Shadow theater exhibit. Chi square analyses were run to check for the gender differences in noticing the Shadow theater and to see if the families with an experimentation activity prompt were more likely to notice the Shadow theater. There were no gender differences in engaging with the exhibit [*χ*^2^(1, *N* = 39) = 3.49, *p* = 0.09], neither were the families with the prompt more likely to notice the Shadow theater [*χ*^2^(1, *N* = 39) = 0.24, *p* = 0.62]. Fifteen families also experimented with the shadow and explained the effects. Chi square analyses were run to see if receiving the experimentation activities prompt was related to the experimentation. Receiving the prompt was related to the experimentation [*χ*^2^(1, *N* = 39) = 17.25, *p* < 0.001]. Seventy percent of the families who got the experimentation activity prompt (14/20) compared to 5% of the families who did not get the prompt (1/19) engaged in experimentation and explanation of the effects at the Shadow theater.

## Discussion

The present study investigated children’s learning experience in a science center with the aim to establish how the different learning behaviors relate to family motivation for the visit and the on-the-spot activities. The hypothesis proposed that families with a learning motivation would engage in more teaching and learning behaviors. Indeed, the results indicated that parents, who reported a motivation to learn something new, asked more open-ended questions and responded to their children’s questions more than parents reporting other motivations for the visit. In addition, it was expected that families with the experimentation activity prompt would engage more with the particular exhibit. Indeed, families receiving a prompt to experiment were more likely to do so compared to families who did not receive such a prompt.

Prior research has revealed open-ended questions and responses to such questions to be important mechanisms for memory formation especially for young children (Hedrick et al., 2009a,b). Questions and answers are also a common pedagogical device (Cotton, 1988). In the present study, learning motivation was related to parental questions and responses. Therefore, it seems that adults recognize questions and answers as an inherent learning mechanism and engage in them more if their aim is to gain or provide new knowledge. This is reassuring, on the one hand, as parents with a learning motivation seem to be acting in a way that supports their children’s learning and memory. On the other hand, there is reason to contemplate how to help parents recognize other conversational devices (e.g., making associations) as beneficial for children’s learning. There are data to suggest that after a certain level of expertise is acquired by children, questions that parents pose act as tests of that knowledge rather than mechanisms to move the knowledge further (Palmquist and Crowley, 2007).

A wide variety of methods may be used to prompt deeper engagement, i.e., experimentation or prolonged discussion of the phenomena in informal learning contexts (e.g., Braham et al., 2018; Callanan et al., 2020; Land-Zandstra et al., 2020). Prior research has indicated that on-the-spot activities help children to experience more conversation on the topic (Tenenbaum et al., 2010) and remember the experience better (Jant et al., 2014). The results of the present study demonstrated a relationship between receiving an experimentation activity prompt and active experimentation and deeper exploration of the concept. At the same time, the prompt was not related to positive changes in family learning interaction that would generalize over the whole visit. On the contrary, children with the experimentation activity prompt were less likely to use associations and explanations.

Hence, using prompts in the form of worksheets in a hands-on exhibit seems to be a double-edged sword. On the one hand, the families with the prompt clearly engaged in more experimentation and explored the concept of shadow to a larger extent than families without the prompt. On the other hand, children with the prompt were less likely to use associations and explanations and consequently engaged with other exhibits less at a verbal level. This could be a sign of concern as it could indicate that the prompt in the form of a worksheet distracts them from fully focusing on the exhibits and engaging with them on a deeper cognitive level. Indeed, it was observed from the videos that sometimes children had trouble with carrying the worksheet along and did not know where to put it when engaging with the hands-on exhibits.

Therefore, the question for the informal education specialists remains how to make experimentation an integral part of the exhibit. Several studies indicate that building interactive exhibits that invite iteration and experimentation engage parents and children in science and engineering learning talk (Tscholl and Lindgren, 2016; Pagano et al., 2020). Interactive exhibits without these qualities could hinder interaction and learning (Heath et al., 2005). With some exhibits like the Shadow theater, in the case of the present study, the opportunity for experimentation could be less eminent. Researchers have successfully incorporated open-questions in the signage to boost metacognition (Gutwill and Dancstep, 2017) and family conversations (Land-Zandstra et al., 2020). Perhaps integrating questions in the exhibit or signage could also prompt experimentation in a hands-on exhibit.

It is worth noting that the study revealed a gender difference in the parent interactions based on the gender of the child with parents more likely to explain to girls. A few studies have reported that parents talk to girls and boys differently when discussing science related topics (Crowley et al., 2001b; Tenenbaum and Leaper, 2003; Tenenbaum et al., 2005). These studies have generally pointed out that parents talk to and explain to girls less as compared to boys when the topic is science related. The present study found evidence to the contrary. In addition, gender was associated to one children’s conversational variable: girls were likely to use more attention directing than boys. It is possible that these findings are related to child behavior in the science center in general. The present study did not focus on the time children spent in the company of their parents and the time they explored alone. Yet, based on the impression from the videos, it is possible that boys were more likely to wonder off and explore by themselves and, therefore, possibly spent less time in the company of their parents. This could be the reason some of them did not point different exhibits out to the parents as often and were simply not around to hear explanations. This aspect should be addressed in more detail in future studies.

There are several limitations to the study. The sample is rather small and does not allow for the thorough investigation of three way interactions including, for example, child gender. In addition, the study focused on parent reported motivation for the visit and not children’s motivation. Children may have their own agendas that guide their visit (Anderson et al., 2008). Motivation was inquired at the end of the visit; hence, it is possible that the experience of the visit itself guided the selection of motives to some extent. Whether this is possible, should be addressed in larger visitor motivation studies. Other factors, such as parental prior knowledge (Franse et al., 2020) and parents’ ideas about if and how children should gain knowledge from such visits (Gaskins, 2008) could guide their activities and conversations with their children. These aspects should be studied alongside motivation for the visit in the future.

Nevertheless, the study provides an understanding how parental motivation is linked to their conversation with their children in a science center and shows how an on-the-spot activity could shape the family visit. These findings carry several implications. First, science centers and other informal learning environments (such as zoos and aquariums) are well-established as locations for family recreational activities. It may be useful to try and activate the learning motivation of the visiting families as it may bring about conversations that create more learning opportunities for children. In a similar vein, creating circumstances that would allow parents to make associations and to provide explanations with ease might take children’s learning opportunities even a step further. Second, the results also imply that it is important to choose fitting on-the-spot interventions, and it may be useful to integrate suggestions to experiment in the exhibits rather than use worksheets in hands-on science centers. This could also provide a guide for parents who otherwise may fail to provide children with learning opportunities *via* explanations and associations due to lack of knowledge or museum fatigue (Allen, 2004).

## Data Availability Statement

The raw data supporting the conclusions of this article will be made available by the authors, without undue reservation.

## Ethics Statement

The studies involving human participants were reviewed and approved by Research Ethics Committee of the University of Tartu. Written informed consent to participate in this study was provided by the participants’ legal guardian/next of kin.

## Author Contributions

The author confirms sole responsibility for the following: study conception and design, data collection, analysis and interpretation of results, and manuscript preparation.

### Conflict of Interest

The author declares that the research was conducted in the absence of any commercial or financial relationships that could be construed as a potential conflict of interest.
